# Redirected Stress Responses in a Genome-Minimized ‘midi*Bacillus*’ Strain with Enhanced Capacity for Protein Secretion

**DOI:** 10.1128/mSystems.00655-21

**Published:** 2021-12-14

**Authors:** Rocío Aguilar Suárez, Minia Antelo-Varela, Sandra Maaß, Jolanda Neef, Dörte Becher, Jan Maarten van Dijl

**Affiliations:** a University of Groningen, University Medical Center Groningen, Groningen, the Netherlands; b University of Greifswald, Institute of Microbiology, Department of Microbial Proteomics, Greifswald, Germany; Northwestern University

**Keywords:** *Bacillus subtilis*, genome minimization, proteomics, protein secretion, Sec pathway, signal peptidase

## Abstract

Genome engineering offers the possibility to create completely novel cell factories with enhanced properties for biotechnological applications. In recent years, genome minimization was extensively explored in the Gram-positive bacterial cell factory Bacillus subtilis, where up to 42% of the genome encoding dispensable functions was removed. Such studies showed that some strains with minimized genomes gained beneficial features, especially for secretory protein production. However, strains with the most minimal genomes displayed growth defects. This focused our attention on strains with less extensive genomic deletions that display close-to-wild-type growth properties while retaining the acquired beneficial traits in secretory protein production. A strain of this category is B. subtilis IIG-Bs27-47-24, here referred to as midi*Bacillus*, which lacks 30.95% of the parental genome. To date, it was unknown how the altered genomic configuration of midi*Bacillus* impacts cell physiology in general, and protein secretion in particular. The present study bridges this knowledge gap through comparative quantitative proteome analyses with focus on protein secretion. Interestingly, the results show that the secretion stress responses of midi*Bacillus*, as elicited by high-level expression of the immunodominant staphylococcal antigen A, are completely different from secretion stress responses that occur in the parental strain 168. We further show that midi*Bacillus* has an increased capacity for translation and that a variety of critical Sec secretion machinery components is present at elevated levels. Altogether, our observations demonstrate that high-level protein secretion has different consequences for wild-type and genome-engineered *Bacillus* strains, dictated by the altered genomic and proteomic configurations.

**IMPORTANCE** Our present study showcases a genome-minimized nonpathogenic bacterium, the so-called midi*Bacillus*, as a chassis for the development of future industrial strains that serve in the production of high-value difficult-to-produce proteins. In particular, we explain how midi*Bacillus*, which lacks about one-third of the original genome, effectively secretes a protein of the major human pathogen Staphylococcus aureus that cannot be produced by the parental Bacillus subtilis strain. This is important, because the secreted S. aureus protein is exemplary for a range of targets that can be implemented in future antistaphylococcal immunotherapies. Accordingly, we anticipate that midi*Bacillus* chassis will contribute to the development of vaccines that protect both humans and livestock against diseases caused by S. aureus, a bacterial pathogen that is increasingly difficult to fight with antibiotics, because it has accumulated resistances to essentially all antibiotics that are currently in clinical practice.

## INTRODUCTION

Recent advances in genetic engineering and synthetic biology, combined with next-generation sequencing technologies, have allowed the construction of microorganisms with significantly reduced genomes (1–4). Initially, genome reduction was performed to determine the minimal set of genes required for cell growth and viability under favorable conditions and to elucidate the functions of essential genes (3, 5–7). Besides answering these fundamental scientific questions, genome reduction has also offered the possibility to create completely novel strains that can be applied as cell factories in bioproduction processes (8–13).

Bacteria of the genus *Bacillus* have been used extensively in industrial biotechnology as cell factories for protein production, which relates to their high protein secretion capacities and the fact that they are nonpathogenic to humans, animals, and plants (14, 15). Among these bacilli, the Bacillus subtilis strain 168 gained popularity not only because of its application potential but also because it has been an important model organism for studies on Gram-positive bacteria in general. Accordingly, B. subtilis 168 was one of the first organisms with a completely sequenced genome (16). Subsequently, this bacterium became the starting point for extensive genome-wide studies on gene function, involving the individual deletion of all nonessential genes (5). Based on the gathered knowledge, and taking advantage of the excellent genetic amenability of B. subtilis, extensive genome minimization studies were undertaken. This culminated in the engineering of B. subtilis strains that have the largest genome reductions thus far described for any living species (4, 17). One of these massively genome-minimized strains is the so-called ‘mini*Bacillus*’, which lacks ∼35% of the genome of its parental strain 168 (17, 18). However, since minimization of the B. subtilis genome was based on a sequential stepwise process, a host of intermediate genome-reduced strains was created, such as the landmark strains IIG-Bs27-24 and IIG-Bs24-47-24 (17).

An intriguing question that we recently addressed was whether any of the genome-minimized B. subtilis strains, such as mini*Bacillus*, could be applied as a cell factory. Indeed, it was shown that mini*Bacillus* displayed an enhanced capability to produce difficult-to-produce proteins, as was demonstrated by the secretion of several staphylococcal antigens that could not be secreted by the parental strain B. subtilis 168. This was partly explained by the deletion of genes for extracellular proteases and an enhanced capacity for translation in mini*Bacillus*, but overall, the reasons underlying the observed improvements in protein secretion remained unclear (18). Importantly, mini*Bacillus* also displayed some counterproductive traits in the sense that its growth on rich medium was slower than that of the parental strain 168 and that lower cell densities were reached. This suggested that, at one or more stages during the construction of the genome-minimized B. subtilis strains, certain beneficial traits of the parental strain 168 had been lost, while the new beneficial properties were gained. Consistent with this view, we recently described the supersecreting midi*Bacillus* strain (originally designated IIG-Bs24-47-24), which still shows close-to-parental growth properties (19).

Our present study was aimed at investigating in which molecular aspects the midi*Bacillus* strain differs from the parental strain 168, apart from the fact that it lacks 30.95% of the genome, representing 1,401 genes. Among these deleted genes were those that were previously designated dispensable and unwanted, especially prophage-carried genes, the genes for eight major extracellular proteases, and genes involved in sporulation and biofilm formation (20). An additional objective of our present study was to chart the cellular responses to induced high-level production of a secreted heterologous protein in midi*Bacillus* and to compare them with the respective responses of the parental strain 168. To achieve these objectives, we applied a comparative proteomics approach, where the total protein complement of the two strains was compared prior to and during induced production of the immunodominant staphylococcal antigen A (IsaA). In particular, IsaA is a cell-wall-associated and secreted protein from the livestock-associated and human pathogen Staphylococcus aureus. Currently, S. aureus represents a global health concern due to its acquired antibiotic resistances, as critically underscored by the methicillin-resistant S. aureus (MRSA) lineages that have emerged in hospitals and the community. One strategy to combat antibiotic resistance, a top-10 threat to global health (21), is vaccination where specific bacterial antigens can be used for immunization (22). However, there is currently no vaccine against MRSA infections, and the search for suitable staphylococcal antigens for vaccination is still in progress (23, 24). Importantly, the model protein IsaA employed in our present study to asses protein secretion by midi*Bacillus* is a potential target for innovative antistaphylococcal immunotherapy (25–27).

Briefly, in the present study we separately analyzed the protein content of the cytosolic and membrane compartments of IsaA-producing or nonproducing midi*Bacillus* cells and the parental strain 168, as well as their respective extracellular proteins in the growth medium. The concept behind this comprehensive proteome analysis was that the results would disclose the major physiological alterations in midi*Bacillus* that have led to its improved performance in protein production and secretion. Interestingly, the present investigations uncover major rearrangements in the responses of midi*Bacillus* to the protein secretion stress caused by induced IsaA production. These include an upregulation of proteins controlled by the stringent response and a downregulation of various stress-responsive systems. Importantly, compared to the parental strain 168, midi*Bacillus* displays elevated levels of various Sec secretion machinery components. Together, the observed alterations provide novel explanations for the enhanced performance of midi*Bacillus* in heterologous protein secretion.

## RESULTS

### Protein abundance.

**FIG 1:**
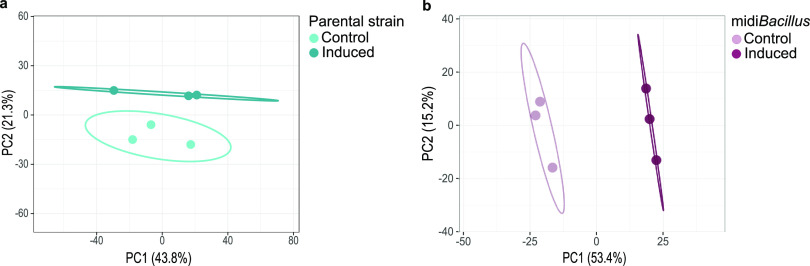
Principal-component analysis of the LFQ protein intensities in the 168 and midi*Bacillus* strains. PCA of the LFQ protein intensities determined for the control and induced conditions in the parental strain 168 (a) and midi*Bacillus* (b). Confidence ellipses are indicated.

A comparative label-free protein quantification was performed to investigate the physiological adaptations of midi*Bacillus* and its parental strain 168 upon production of IsaA. Both strains carrying the plasmid pRAG3::*isaA* for subtilin-inducible expression of IsaA were grown until the exponential growth phase and then induced with subtilin for IsaA production. After 2 h of induction, protein synthesis and abundance were measured and compared to the controls, which were treated in the same way but without subtilin induction. The subsequent liquid chromatography-mass spectrometry (LC-MS) analyses of samples from the parental strain yielded 1,276 and 1,390 uniquely identified proteins for the control and induced conditions, respectively, considering all protein fractions. For midi*Bacillus*, 1,297 and 1,189 different proteins were uniquely identified for the control and induced conditions, respectively. Proteins qualified for subsequent quantification only if they were present in two out of three replicates and localized in the predicted cell fraction. Considering that the 168 and midi*Bacillus* strains are far from isogenic due to the massive genome reduction, the first step in the analysis was to determine the changes in each strain after induction. Subsequently, we assessed whether the observed responses differed between the two strains. The quality of biological replicates was evaluated by a principal-component analysis (PCA). This showed that, for both the 168 and midi*Bacillus* strains, the individual replicates of the induced condition clustered together and were separated from the respective noninduced replicates (Fig. 1). This clearly showed that induction of IsaA production affected the proteomes of both investigated strains.

**FIG 2:**
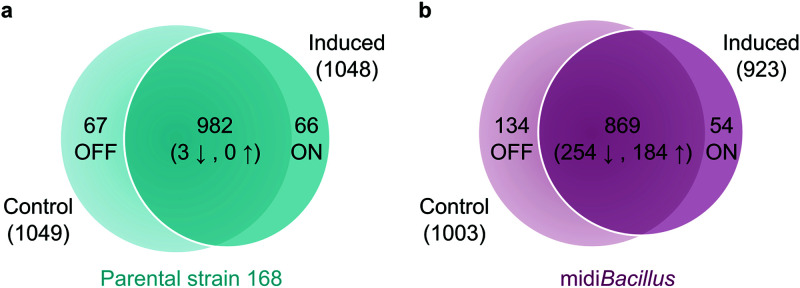
Numbers of proteins that are either unique or shared between conditions. Numbers of significantly upregulated (↑) and downregulated (↓) proteins per condition are presented at the heart of the Venn diagram in the parental strain 168 (a) and midi*Bacillus* (b). Proteins detected only under the noninduced condition are indicated as OFF, and proteins detected exclusively under the induced condition are labeled as ON.

Venn diagrams were used to compare the numbers of quantified proteins of each strain (Fig. 2; see also Fig. S1a in the supplemental material). Proteins that were detected only in the induced cells were designated ‘ON’, and if they were present exclusively in the control, they were designated ‘OFF’. As shown with the Benjamini-Hochberg false-discovery rate (FDR) method, three proteins were significantly downregulated upon IsaA induction in the parental strain, while 254 and 184 proteins were down- or upregulated, respectively, in midi*Bacillus*. These values, together with the numbers of OFF or ON proteins (Fig. 2; Table S2), resulted in a total of 136 and 626 proteins that were altered upon induction of IsaA production in the 168 and midi*Bacillus* strains, respectively.

10.1128/mSystems.00655-21.1 FIG S1 Venn diagram of the quantified proteins under both strains and conditions and box plot of shared proteins between conditions. (a) Proteins were considered for quantification only if they were present in at least 2 of the 3 replicates and identified with a minimum of 2 unique peptides. White numbers indicate the quantified proteins that were detected under both conditions and exclusively in one strain. To note, both strains shared a core of 702 proteins that were expressed under both conditions. (b) Box plot of changes in the amounts of all quantified proteins present under both conditions per functional category. The changes correspond to the proteins in the overlapping region of the Venn diagram. For each protein, the difference was calculated as the amount based on LFQ values in the induced cells minus the values from the noninduced cells. Therefore, a positive value is the result of higher protein abundance under the induced condition than in the control. To note, some proteins belong to multiple functional categories. Outliers (values smaller or larger than the median ± 1.5 times the interquartile range) are plotted as points after the end of the whiskers, bars represent median values, the subdivision of boxes represents quartiles, and whiskers show the upper and lower quartiles. Elements depicted in blue correspond to the parental strain, while elements for midi*Bacillus* are depicted in orange. Download FIG S1, PDF file, 0.3 MB. Copyright © 2021 Aguilar Suárez et al. 2021 Aguilar Suárez et al. https://creativecommons.org/licenses/by/4.0/ This content is distributed under the terms of the Creative Commons Attribution 4.0 International license.

10.1128/mSystems.00655-21.5 TABLE S1 Plasmids and strains used in this study. Download Table S1, XLSX file, 0.01 MB. Copyright © 2021 Aguilar Suárez et al. 2021 Aguilar Suárez et al. https://creativecommons.org/licenses/by/4.0/ This content is distributed under the terms of the Creative Commons Attribution 4.0 International license.

10.1128/mSystems.00655-21.6 TABLE S2 Listed number of identified, quantified, and significantly changed proteins per cell fraction in the B. subtilis 168 strain and midi*Bacillus*. The total numbers of identified and quantified proteins of the noninduced and induced 168 or midi*Bacillus* strains are presented. Download Table S2, XLSX file, 0.01 MB. Copyright © 2021 Aguilar Suárez et al. 2021 Aguilar Suárez et al. https://creativecommons.org/licenses/by/4.0/ This content is distributed under the terms of the Creative Commons Attribution 4.0 International license.

### Proteomic patterns in the parental strain 168 and midi*Bacillus*.

**FIG 3:**
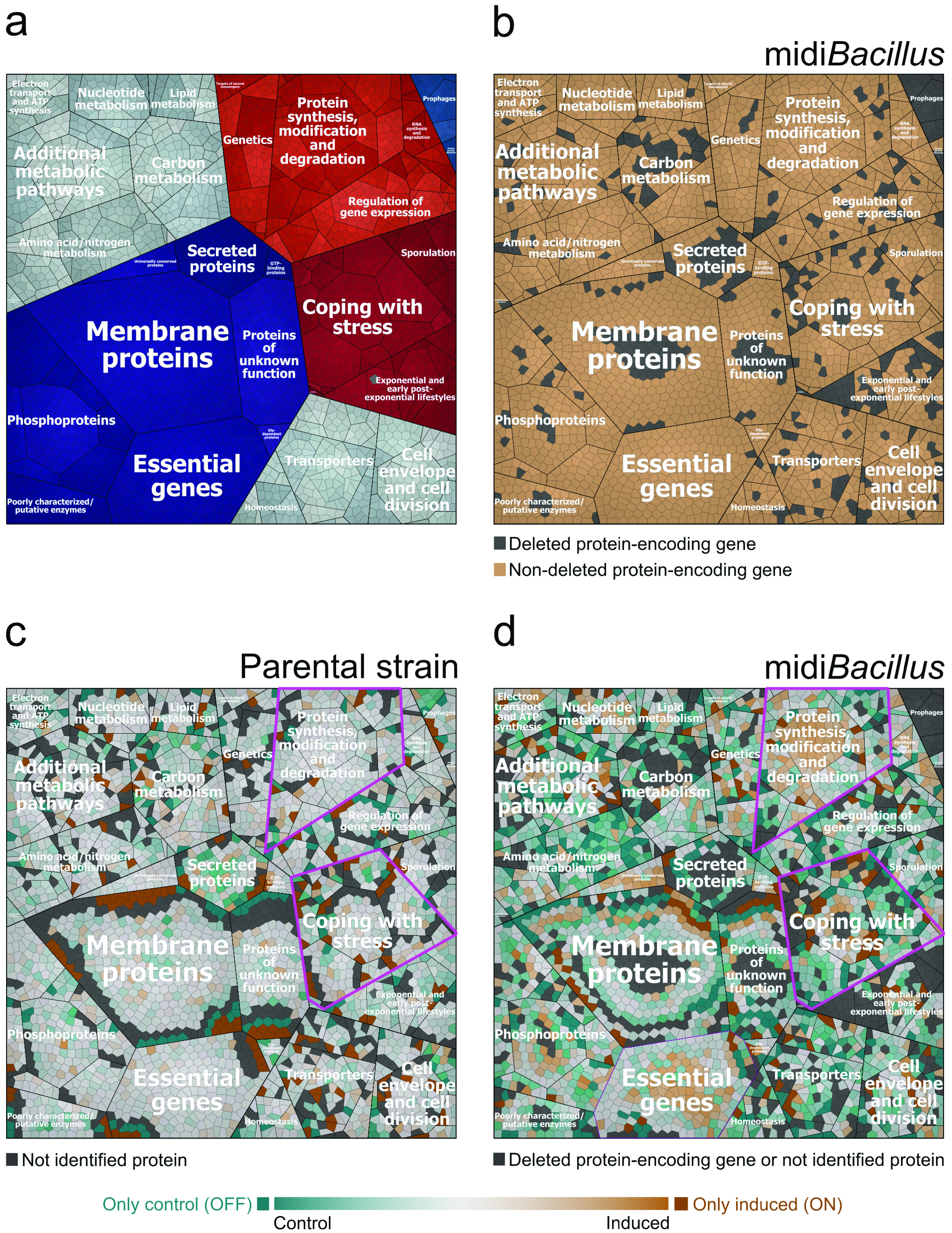
Voronoi treemaps of the quantified proteins in the parental strain 168 and midi*Bacillus* clustered per functional category according to the SubtiWiki database. (a) Functional classification of all quantified proteins of the parental strain and midi*Bacillus* according to the second level of the functional categories defined in SubtiWiki. (b) Designation in color code of quantified proteins for which the protein-encoding genes are absent (colored light gray) or present (colored light brown) in midi*Bacillus*. (c and d) Treemaps of quantified proteins depicting the ratio of protein expression of the control versus the induced condition for the parental strain (c) and midi*Bacillus* (d) are indicated by color code. In panels c and d, proteins colored in shades of orange are more abundant under the induced condition and proteins colored in shades of turquoise are more abundant under the control condition. Furthermore, darker colors illustrate larger differences in protein expression. ON proteins (colored in dark orange) are present only under the induced condition, while OFF proteins (colored in dark turquoise) are present only under the control condition. Nonidentified proteins and proteins encoded by deleted genes are indicated in light gray. To distinguish between proteins of which the genes were deleted or present, please refer to panel b. Relevant functional groups are highlighted by fuchsia-colored outlines in panels c and d.

To identify the processes in which the quantified proteins of the 168 and midi*Bacillus* strains were involved, functional categories were assigned to the respective proteins according to the SubtiWiki database (28) (Table S3). Differences in protein amounts between the noninduced and induced cultures were then visualized in Voronoi treemaps according to their functional category. To this end, functionally related gene products were assigned to the same cluster and partitioned into weighted polygons with an area proportional to the relative weight of the respective functional category (Fig. 3). Details of further subdivision of the functional categories up to the level of protein names are provided in Fig. S2. Here, it should be noted that missing values of lower levels of the functional annotation categories were supplemented with the information available from the higher annotation levels. The resulting Voronoi treemaps thus consider all the quantified proteins of both strains and facilitate a visual comparison. Additionally, the proteins encoded by genes deleted from midi*Bacillus* are also indicated in the treemaps (Fig. 3c and Fig. S2c). In general, the difference in the expression of proteins between conditions is broader in midi*Bacillus* than in the parental strain, where most of the values oscillated from −0.5 to 0.5 (Fig. S1b). Differences in the protein patterns of the two strains are especially evident for universally conserved proteins, proteins involved in protein synthesis and modification, proteins that are encoded by essential genes, and proteins involved in the biosynthesis and acquisition of nucleotides (Fig. 3; Fig. S2). In fact, most of the proteins in these categories were upregulated in midi*Bacillus* upon induced production of IsaA (Table S3 and Fig. S3). Here, it is especially noteworthy that this upregulation was significant for ribosomal proteins, belonging to the first two specified categories. Meanwhile, for the parental strain an increased abundance was observed in particular for proteins that are associated with mobile genetic elements, representing more than 2 quartiles of the identified proteins in this category (Fig. S1b). The functional protein categories including higher numbers of downregulated and OFF proteins in midi*Bacillus* were membrane proteins and proteins coping with stress and amino acid nitrogen metabolism. In the parental strain 168, downregulated and OFF proteins were membrane proteins and proteins involved in the regulation of gene expression. Altogether, the two strains shared a core of 702 proteins that were quantified under both conditions (Fig. S1a), of which 24.5% are involved in protein synthesis and modification (Table S4).

10.1128/mSystems.00655-21.2 FIG S2 Voronoi treemaps of the quantified proteins in the parental strain and midi*Bacillus* clustered per functional categories according to the SubtiWiki database. The first panel shows level three of the functional categories, level four is indicated in the second panel, and protein names are presented in the last panel. (a and b) Treemaps present the ratio of protein expression of the control to that under the induced condition, for the parental strain (a) and midi*Bacillus* (b), as color code. Proteins colored in shades of orange are more abundant under the induced condition; proteins colored in shades of turquoise are more abundant under the control condition. Therefore, darker colors illustrate larger differences in protein expression. ON proteins (colored in dark orange) are present only under the induced condition, while OFF proteins (colored in dark turquoise) are present only under the control condition. In light gray color are indicated the nonidentified proteins in panel a and the nonidentified or deleted protein-encoding genes in panel b. Relevant regulons are highlighted by fuchsia-colored outlines in panels a and b. (c) For all quantified proteins of the parental strain and the midi*Bacillus* strain, it is indicated whether the respective protein-encoding gene is deleted from (in light gray color) or present in (light brown) the midi*Bacillus* genome. Download FIG S2, PDF file, 1.1 MB. Copyright © 2021 Aguilar Suárez et al. 2021 Aguilar Suárez et al. https://creativecommons.org/licenses/by/4.0/ This content is distributed under the terms of the Creative Commons Attribution 4.0 International license.

10.1128/mSystems.00655-21.3 FIG S3 Distribution of quantified proteins enriched per functional category. Percentages were calculated based on the total number of proteins in the parental strain per category according to the SubtiWiki database. Proteins in the noninduced control (OFF) and significantly downregulated proteins are represented in the lower part of the bar followed by the equally abundant proteins and, subsequently, the group of ON and significantly upregulated proteins per condition. Proteins whose genes were deleted in the midi*Bacillus* strain are indicated with light gray diagonal lines at the top of the bar. Nonidentified proteins are not depicted for better visualization of the data, but their percentage can be inferred by subtraction of the depicted data from 100%. Of note, the higher total percentage of proteins identified for midi*Bacillus* than for strain 168 means that relatively more proteins of a particular category were identified for midi*Bacillus*. Download FIG S3, PDF file, 0.3 MB. Copyright © 2021 Aguilar Suárez et al. 2021 Aguilar Suárez et al. https://creativecommons.org/licenses/by/4.0/ This content is distributed under the terms of the Creative Commons Attribution 4.0 International license.

10.1128/mSystems.00655-21.7 TABLE S3 Functional categories and LFQ intensities of the quantified proteins. To identify the processes in which the quantified proteins are involved, functional categories were assigned according to the SubtiWiki database. The log transformation of the label-free quantification intensities is given for each replicate, and the differences between conditions were calculated based on the mean values of the biological replicates and represented as log_2_ fold change. The Benjamini-Hochberg FDR method was used to compute multiple testing corrections for *P* values to determine the proteins that changed significantly between conditions. Download Table S3, XLSX file, 1.3 MB. Copyright © 2021 Aguilar Suárez et al. 2021 Aguilar Suárez et al. https://creativecommons.org/licenses/by/4.0/ This content is distributed under the terms of the Creative Commons Attribution 4.0 International license.

10.1128/mSystems.00655-21.8 TABLE S4 Functional category distribution of the core of 702 proteins present in both strains and under both conditions. Note that some proteins are allocated to multiple functional categories. Download Table S4, XLSX file, 0.01 MB. Copyright © 2021 Aguilar Suárez et al. 2021 Aguilar Suárez et al. https://creativecommons.org/licenses/by/4.0/ This content is distributed under the terms of the Creative Commons Attribution 4.0 International license.

### Translation capacity.

**FIG 4:**
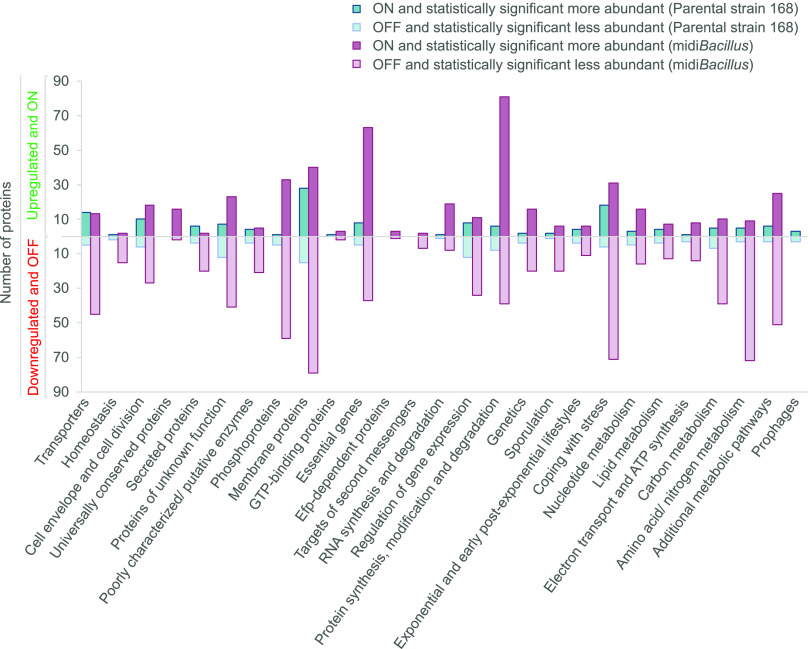
Total numbers of ON, OFF, and significantly regulated proteins upon induction of IsaA production per functional category. In the upper part of the plot, the numbers of upregulated and ON proteins are presented. In the lower part, in a mirror scale, the numbers of downregulated and OFF proteins are presented.

**FIG 5:**
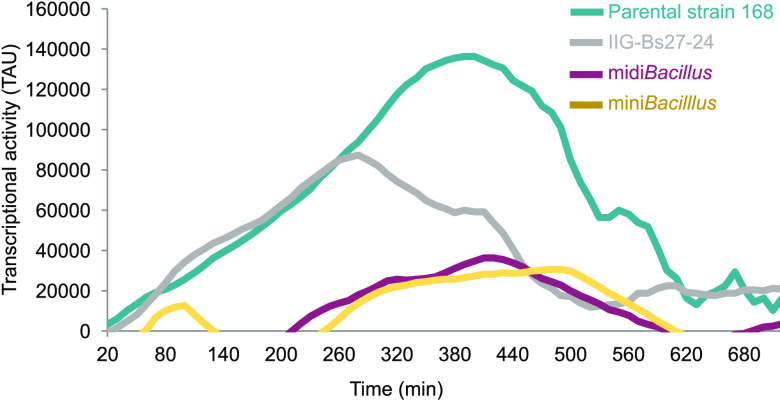
Translational efficiency in the parental strain 168 and its genome-reduced derivatives approximated with a synthetic *bmrC*-GFP expression module. Transcriptional activity of the *bmrCD* genes in different B. subtilis strains was measured in real time using a synthetic *bmrC*-GFP expression module as previously described for the mini*Bacillus* strain (18). Of note, GFP transcriptional activity (TAU) in the four indicated strains was assessed in the presence of 0.2 μg/ml of clindamycin.

The response of midi*Bacillus* to induction of IsaA production is characterized by an increment in proteins that are involved in protein synthesis and modification, as underpinned by the highest number of significantly upregulated and ON proteins (Fig. 4). This finding is in line with our previous study focused on the absolute changes in the membrane proteome upon IsaA induction (19). Importantly, the present data highlight a key difference between midi*Bacillus* and the parental strain, namely, the fact that the translation machinery is boosted only in midi*Bacillus*. Specifically, 90% of the ribosomal proteins were significantly upregulated in midi*Bacillus*, while 98% of these proteins did not show a change in the parental strain (Table S3). Since we quantified more proteins with translation functions in midi*Bacillus* than in the parental strain, i.e., 150 versus 133 proteins, respectively (Table S3), we decided to evaluate the overall translation rates in both strains. To this end, we used a previously developed synthetic reporter module where green fluorescent protein (GFP) transcription is coupled to expression of the *bmrC* gene of B. subtilis (29). Expression of the *bmrC* and *bmrD* genes, which encode an inducible drug efflux pump, is regulated via transcriptional attenuation, involving the leader peptide BmrB encoded by the *bmrBCD* operon (29). Importantly, efficient translation of *bmrB* results in the formation of a terminator that precludes transcription of *bmrC* and *bmrD*. Conversely, slowed-down translation of BmrB by ribosome-targeted antibiotics, such as clindamycin, will favor the formation of an antiterminator and trigger increased expression of *bmrC* and *bmrD*. Thus, to identify possible differences in overall translation rates, the *bmrC*-GFP fusion was integrated into the *bmrBCD* loci of the midi*Bacillus* and 168 strains. Subsequently, we assessed the levels of GFP expression during growth in the presence of a small amount of clindamycin that triggers *bmrC*-GFP expression with the premise that *bmrC*-GFP expression in midi*Bacillus* will not be strongly induced by clindamycin if overall the translation rate is high (18, 29). Consistent with the proteomics data, the expression of GFP in midi*Bacillus* with the *bmrC*-GFP module remained far below that of the parental strain with this module (Fig. 5). This implies a significantly enhanced efficiency of BmrB translation in midi*Bacillus* compared to the parental strain. Interestingly, *bmrC*-GFP expression in midi*Bacillus* was comparable to the previously assessed *bmrC*-GFP expression in the mini*Bacillus* strain PG10 (18) (Fig. 5). On the other hand, it remained significantly lower than the *bmrC*-GFP expression measured in strain IIG-Bs27-24 (17, 18) (Fig. 5), which is a close ancestor of midi*Bacillus* in the phylogeny of genome-minimized B. subtilis strains. These findings imply that midi*Bacillus* displays essentially the same optimal translational capabilities as the previously described mini*Bacillus*.

### Secretion via the Sec pathway.

**FIG 6:**
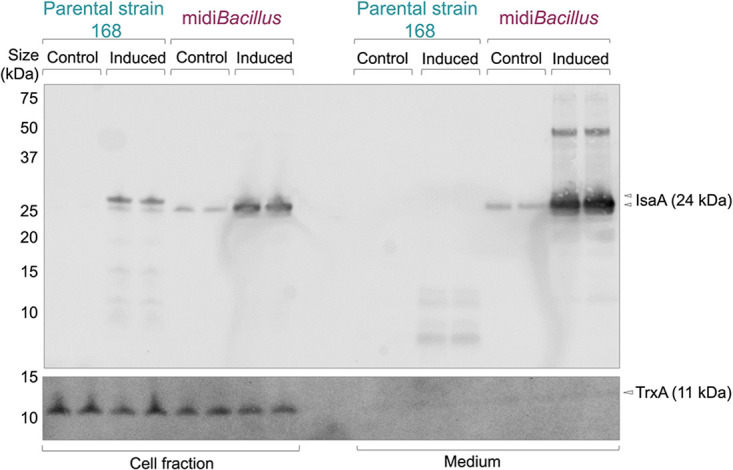
Induced overproduction of the staphylococcal antigen IsaA in the parental strain 168 and midi*Bacillus*. To allow induction of IsaA expression from plasmid pRAG3::*isaA* with subtilin, the *spaRK* genes were introduced in the *amyE* locus of the midi*Bacillus* and 168 strains. Culture samples were collected 2 h after induction with subtilin. At the same time, samples were withdrawn from parallel noninduced cultures. Cells were separated from the growth medium by centrifugation, and proteins in the respective fractions were analyzed by LDS-PAGE and Western blotting with the IsaA-specific monoclonal antibody 1D9. The cytoplasmic marker protein for cell lysis TrxA was detected with a specific polyclonal antibody. The positions of precursor and mature forms of IsaA and the TrxA protein are marked with arrowheads. Of note, precursor and mature forms of IsaA are distinguished based on the fact that the designated precursor form is exclusively identified in the cell fraction, has a lower mobility on LDS-PAGE, and shows a deduced *M*_w_ difference with the secreted mature protein that is consistent with the presence of a signal peptide.

**FIG 7:**
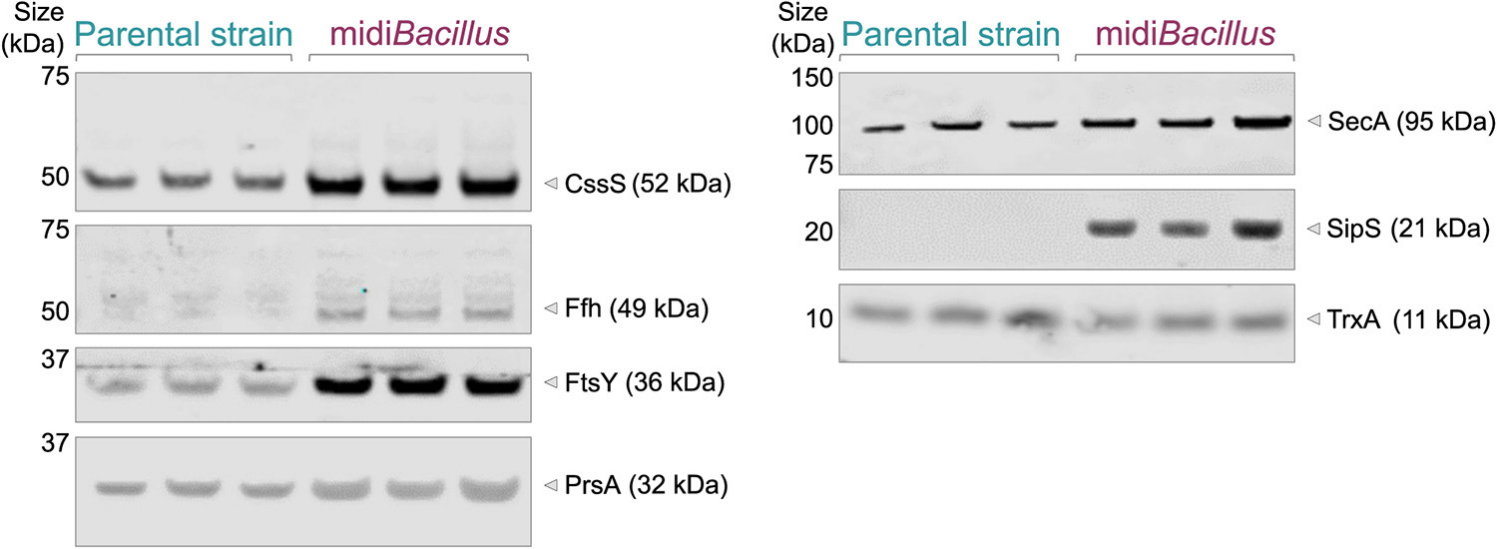
Levels of Sec pathway components in the B. subtilis 168 and midi*Bacillus* strains. The expression of IsaA by B. subtilis 168 and midi*Bacillus* was induced with subtilin, and culture samples were collected 2 h after induction. The cellular levels of CssS, SRP/Ffh, FtsY, PrsA, SecA, SipS, and the cytoplasmic control protein TrxA were assessed by Western blotting with specific polyclonal antibodies. Samples from three biological replicate cultures of each strain were loaded on the gels.

IsaA was significantly upregulated in the extracellular fraction of midi*Bacillus* [−Log(*P* value) = 3.025, difference = 3.710], and it was also detected by MS in the extracellular fraction of the induced parental strain 168 (Table S3). However, since the MS data do not provide information about the integrity of the secreted IsaA protein, we corroborated the abundance of IsaA in the extracellular fractions of the 168 and midi*Bacillus* strains by Western blotting (Fig. 6). In addition, the presence of IsaA in the cell fractions was assessed using the same approach. Indeed, full-size IsaA was detectable in the induced cells of both strains. Judged by a slightly lower mobility on lithium dodecyl sulfate (LDS)-PAGE, it seems that IsaA accumulated in a precursor form in cells of the parental strain, whereas IsaA in the midi*Bacillus* cells was apparently present in a processed mature form (Fig. 6). Similar findings were previously reported for the mini*Bacillus* strain overproducing IsaA (18). Importantly, mature IsaA was abundantly secreted by midi*Bacillus*, whereas predominantly degradation fragments of IsaA were detected in the extracellular fraction of strain 168 (Fig. 6). The detection of enhanced amounts of intact IsaA secreted by midi*Bacillus* can be attributed to reduced protease activity, since this strain lacks eight major secreted proteases (17). However, this does not explain the improved secretion of IsaA compared to the parental strain. One possible reason could be that the genome reduction impacted the quantity of secretion machinery components in midi*Bacillus* compared to the 168 strain. Of note, it is difficult to quantify differences between these two strains from the proteomics data due to the massive differences they display. Therefore, we first compared the proteome data for the induced and noninduced conditions per strain. This showed that induction *per se* did not change the levels of the majority of secretion machinery components (Table 1). Based on the proteomic analysis, only Rnc, a RNase III with different regulatory functions in protein synthesis, modification, and secretion, showed significant upregulation in midi*Bacillus* upon IsaA induction (Table 1). To directly compare the levels of secretion machinery components in the midi*Bacillus* and 168 strains, we performed a Western blot analysis, where equal OD_600_ (optical density at 600 nm) equivalents of the respective induced cultures were used for gel loading (Fig. 7). The relative protein levels were then calculated by ImageJ analysis, and the relative numbers in the parental strain were considered 1. As shown by two-tailed *t* tests, indeed, statistically significant changes in the relative amounts of Sec secretion machinery components had occurred in midi*Bacillus*. In particular, elevated levels of the signal recognition particle (SRP)-related components Ffh (9.0-fold, standard deviation [SD] ±1.1, *P* < 0.01) and FtsY (4.5-fold, SD ±0.4, *P* < 0.01), the signal peptidase SipS (not detected in the parental strain), and the posttranslocational protein folding catalyst PrsA (1.4-fold, SD ±0.2, *P* < 0.05) were detected in midi*Bacillus*. The relative values for the parental strain 168 were 1, SD ±0.3, for Ffh; 1, SD ±0.1, for FtsY; and 1, SD ±0.08, for PrsA. In particular, the strongly enhanced level of SipS would, by itself, be sufficient to explain the observed improvement in IsaA precursor maturation and secretion as shown in Fig. 6, because it was previously shown that overproduction of SipS leads to enhanced signal peptide processing in secretory precursor proteins (30). However, also the enhanced levels of the SRP-related proteins (Ffh, FtsY) and PrsA could very well contribute to improved IsaA secretion (Fig. 7) (31, 32). In addition, a minor increase in the cellular level of the translocation ATPase SecA was observed, which could contribute to improved IsaA secretion as well.

**TABLE 1:** Regulation of quantified proteins involved in the general protein secretion (Sec) pathway and the response to protein secretion stress^*a*^

Protein	Parental strain 168			midi*Bacillus*		
	−log(*P* value)	Difference	Regulation	−log(*P* value)	Difference	Regulation
CssS	NA	NA		0.968	−0.157	=
EcsA	0.016	0.015	=	0.444	−0.181	=
EcsB	0.468	0.245	=	0.394	−0.2	=
HtrB	NA	NA	ON	NA	NA	OFF
Ffh	0.855	0.286	=	0.746	0.206	=
FlhA*	0.181	−0.162	=			
FtsY	0.02	0.01	=	1.968	−0.394	↓
PrsA	0.653	0.372	=	0.353	−0.195	=
RasP	0.054	−0.041	=	1.507	−0.310	=
Rnc	0.067	−0.021	=	3.547	0.82	↑
SecA	0.735	−0.106	=	0.488	−0.115	=
SecDF	0.06	−0.045	=	0.318	−0.109	=
SecG	NA	NA		0.177	−0.169	=
SecY	0.068	0.057	=	0.197	0.103	=
SipS	0.162	0.147	=	1.378	−0.372	=
SipT	0.158	0.141	=	1.37	−0.437	=
SipU	NA	NA		0.104	−0.113	=
SipW*	NA	NA	OFF			
SppA	0.23	0.104	=	1.584	0.443	=
YacD	0.015	−0.011	=	0.416	0.164	=
Yidc1	0.355	0.212	=	0.086	0.054	=

a Abbreviation and symbols: NA, not applied—the protein was not quantified under any condition or only under one condition where the regulation is marked as ON or OFF; *, protein-encoding gene was deleted in midi*Bacillus*; =, difference in the protein abundance was not significant; ↓, the protein was significantly downregulated; ↑, the protein was significantly upregulated.

Another possible reason why IsaA secretion is enhanced in midi*Bacillus* could be that this strain secretes fewer proteins via the Sec pathway, thereby lowering the possible competition for translocation sites. We therefore inspected the extracellular proteome data of the midi*Bacillus* and 168 strains for proteins that are secreted with the help of Sec-type signal peptides using the GP4 algorithm (33). Overall, the number of secreted proteins upon IsaA induction was ∼3-fold higher in the parental strain 168 than in midi*Bacillus*, as judged by the number of ON and significantly upregulated proteins that belong to the functional category of secreted proteins as defined in the SubtiWiki database (see the fifth category from the left in Fig. 4 and Fig. S3). Accordingly, the number of quantified extracellular proteins with GP4-predicted Sec-type signal peptides was also ∼3-fold higher in the parental strain (Table S6a). Furthermore, based on the proteome data, we approximated the proportion of the amounts of proteins secreted via the Sec pathway. As shown by *t* tests, the relative abundance of the proteins secreted via Sec in the extracellular fraction upon IsaA induction was significantly lower in midi*Bacillus* (72.4%, SD ±0.7) than in the parental 168 strain (78.9%, SD ±0.7; *P* < 0.01). Importantly, this lower number of extracellular proteins did not relate to an accumulation of signal peptide-containing proteins in the membrane or cytoplasm of midi*Bacillus*, where, respectively, only two such proteins were identified while remaining undetected in the extracellular fraction (Table S6b). Altogether, these findings demonstrate that in midi*Bacillus* fewer proteins with Sec-type signal peptides compete for the available Sec translocons, which appear to be present in more copies per cell. Nonetheless, the XynA protein of B. subtilis was no longer detected upon IsaA induction, which suggests that IsaA might still be competing with XynA for the Sec pathway, possibly due to substitution of the signal peptide of XynA for the native signal peptide of IsaA.

10.1128/mSystems.00655-21.10 TABLE S6 Prediction of the Sec signal peptides of quantified proteins. GP4 (Gram-positive protein prediction pipeline) was used to predict the signal peptides of the quantified proteins in the extracellular fraction upon IsaA induction in the parental strain and midi*Bacillus* (a) and the extracellular proteins quantified in the cytoplasmic and membrane fractions upon IsaA induction in the parental strain and midi*Bacillus* (b). Download Table S6, XLSX file, 0.01 MB. Copyright © 2021 Aguilar Suárez et al. 2021 Aguilar Suárez et al. https://creativecommons.org/licenses/by/4.0/ This content is distributed under the terms of the Creative Commons Attribution 4.0 International license.

### Secretion stress.

**FIG 8:**
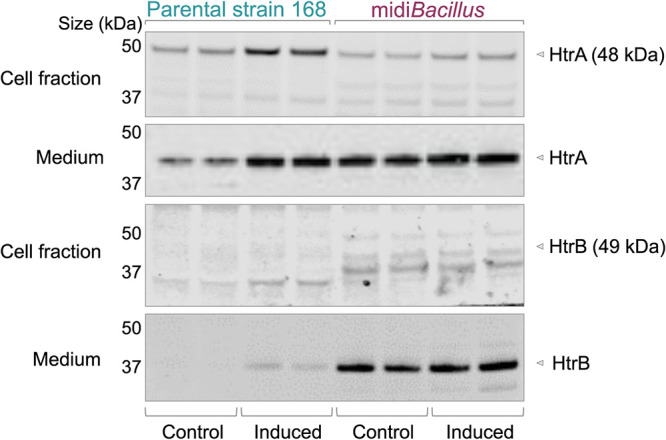
HtrA and HtrB levels in the parental strain 168 and midi*Bacillus*. The expression of IsaA was induced with subtilin, and culture samples were collected 2 h after induction. In parallel, samples were collected from noninduced cultures. Cell-associated and extracellular proteins were separated by LDS-PAGE, and the presence of HtrA and HtrB was assessed by Western blotting with polyclonal antibodies specific for HtrA or HtrB.

B. subtilis cells are known to respond to high-level protein secretion by mounting a so-called secretion stress response, which is dependent on the CssRS two-component regulatory system. Induction of the response results in increased expression of the quality control serine proteases HtrA and HtrB, which also have chaperone activity (32). While HtrA was not detected by the proteome analyses, the HtrB protein was detected upon induced IsaA expression in the parental strain 168 (Table 1). Since this suggested the absence of a secretion stress response in midi*Bacillus*, the levels of HtrA and HtrB were investigated by Western blotting. As expected, the parental strain 168 mounted a typical secretion stress response upon induced expression of IsaA, as both HtrA and HtrB were found to be upregulated (Fig. 8). Here, it should be noted that the full-size induced HtrB was detected only in the extracellular fraction. In contrast, no enhanced expression of HtrA or HtrB was detectable in midi*Bacillus* upon IsaA induction. However, the expression levels of HtrA and HtrB were found to be highly upregulated already in noninduced midi*Bacillus*. In this case, the level of HtrA was comparable to the secretion stress-induced HtrA level in the 168 strain, whereas the extracellular HtrB level was massively increased compared to the 168 strain (Fig. 8). This could mean that noninduced midi*Bacillus* is secretion stressed due to an unintentional aberrant production of one or more secreted proteins. However, a simpler explanation can be found in the enhanced level of the sensor component CssS in midi*Bacillus* (Fig. 7), which might lead to a deregulated response where the noninduced cells are already more perceptive of minor perturbations that trigger the CssRS two-component system.

### A redefined secretion stress response in midi*Bacillus*.

**FIG 9:**
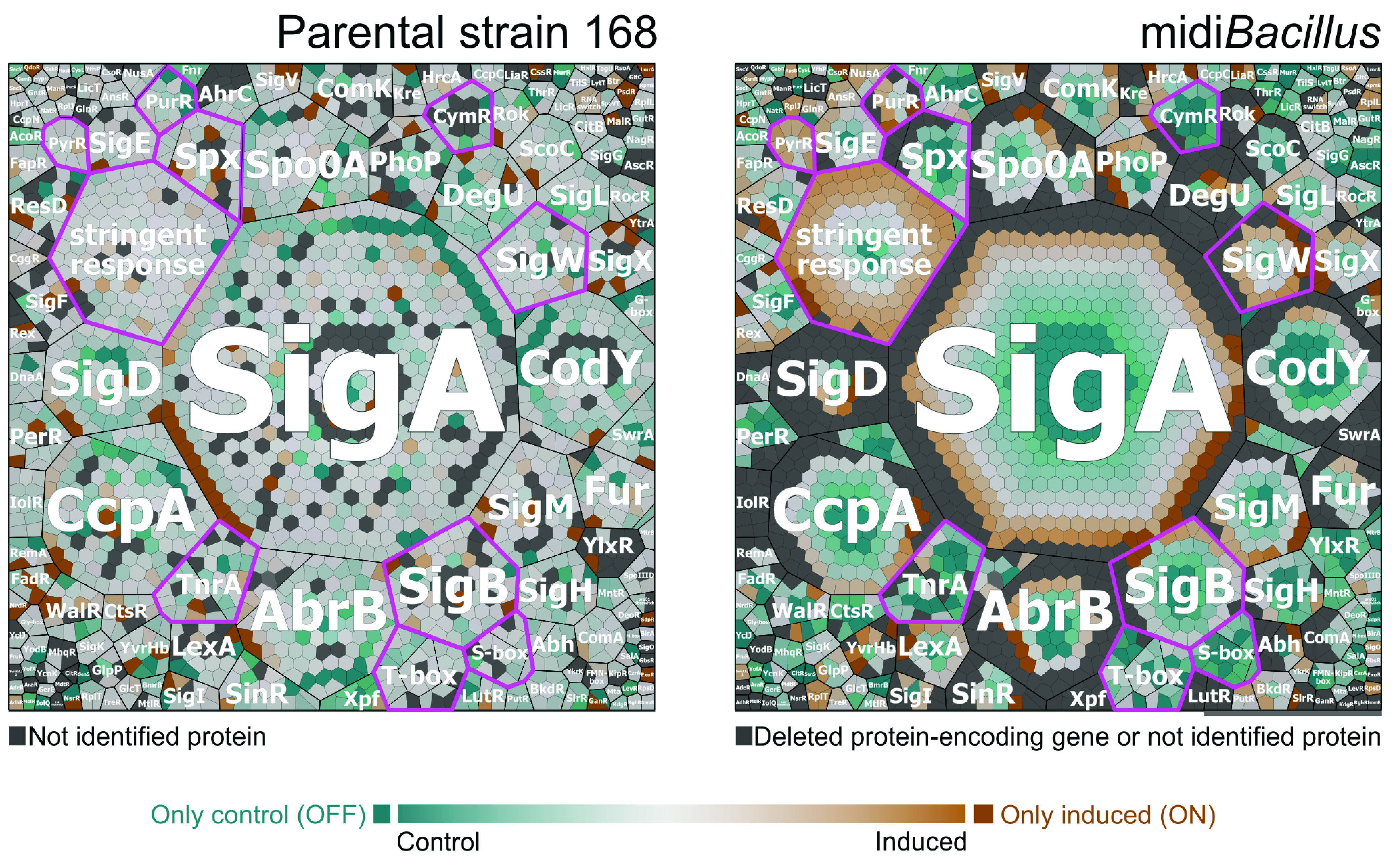
Voronoi treemaps showing the quantified proteins in the parental strain 168 and midi*Bacillus* grouped per regulon. Proteins of B. subtilis 168 (left) and midi*Bacillus* (right) with altered abundance upon IsaA induction were grouped per regulon. The ratio of protein expression under the induced condition versus the noninduced conditions is presented in color code. Proteins colored in shades of orange are more abundant under the induced condition, and proteins colored in shades of turquoise are more abundant under the control condition. Furthermore, darker colors illustrate larger differences in protein expression. ON proteins (colored in dark orange) are present only under the induced condition, while OFF proteins (colored in dark turquoise) are present only under the control condition. Nonidentified proteins and proteins encoded by deleted genes are indicated in light gray. Regulons of interest are highlighted by fuchsia-colored outlines.

An intriguing observation from the functional analysis of proteins regulated upon IsaA induction was that many proteins involved in “coping with stress” were downregulated or no longer detected in midi*Bacillus* (Fig. 3 and 4). In contrast, this was not the case in the parental strain 168. Since this was suggestive of a completely altered perception of protein secretion stress by midi*Bacillus*, we analyzed the respective changes based on regulons. To this end, all quantified proteins in the parental strain and midi*Bacillus* were assigned to regulons according to the SubtiWiki database (28) (Table S5), and Voronoi treemaps were created based on this regulon stratification as shown in Fig. 9. To facilitate comparisons between the two strains, all proteins in these treemaps are presented in the same layout. Additional treemaps displaying the particular modes of regulation, the protein names, and proteins for which the respective genes were deleted in midi*Bacillus* are provided in Fig. S4. Importantly, this analysis highlights two overarching responses of midi*Bacillus* to IsaA induction in terms of protein up- or downregulation. First, the abundance of proteins regulated by the stringent response was highly upregulated. In fact, the majority of quantified proteins of midi*Bacillus* belonging to this regulon were more abundant under the induced condition (Fig. 9). Clearly, this response was not observed in the parental strain 168. The same was true for proteins belonging to the PyrR and PurR regulons for pyrimidine and purine biosynthesis, which were upregulated upon IsaA induction in midi*Bacillus* (Fig. S2). Lastly, proteins belonging to the SigW regulon were more abundant upon IsaA induction in midi*Bacillus*, suggesting that the bacteria perceived some cell envelope perturbations (34, 35). On the other hand, IsaA induction in midi*Bacillus* resulted in a strong downregulation of proteins belonging to the Spx, CymR, SigB, T-box, S-box, and TnrA regulons (Fig. 9) (36–39), suggesting that the bacteria perceived IsaA production as a relatively “relaxing” activity. In contrast, some proteins belonging to the Spx and SigB regulons were upregulated upon IsaA induction in the parental strain 168. Altogether, these data show that the secretion stress response in midi*Bacillus* was completely redefined by this strain’s reconfigured genome.

10.1128/mSystems.00655-21.4 FIG S4 Voronoi treemaps of the quantified proteins in the parental strain and midi*Bacillus* clustered per regulons according to the SubtiWiki database. The first panel shows the regulator of each regulon, the mode of regulation is indicated in the second panel, and protein names are presented in the last panel. (a and b) Treemaps present the ratio of protein expression of the control to that under the induced condition, for the parental strain (a) and midi*Bacillus* (b), as color code. Proteins colored in shades of orange are more abundant under the induced condition; proteins colored in shades of turquoise are more abundant under the control condition. Therefore, darker colors illustrate larger differences in protein expression. ON proteins, which are present only under the induced condition, are colored in dark orange; OFF proteins, which are present only under the control condition, are colored in dark turquoise. In light gray color are indicated the nonidentified proteins in panel a and the nonidentified or deleted protein-encoding genes in panel b. Relevant regulons are highlighted by fuchsia-colored outlines in panels a and b. (c) For all quantified proteins in the parental strain and midi*Bacillus*, it is indicated if the respective protein-encoding gene is deleted from (in light gray color) or present in (light brown) the genome of midi*Bacillus*. Download FIG S4, PDF file, 0.8 MB. Copyright © 2021 Aguilar Suárez et al. 2021 Aguilar Suárez et al. https://creativecommons.org/licenses/by/4.0/ This content is distributed under the terms of the Creative Commons Attribution 4.0 International license.

10.1128/mSystems.00655-21.9 TABLE S5 Quantified proteins regulated by regulons. Quantified proteins that are regulated by regulons were clustered according to the SubtiWiki database. The log transformation of the label-free quantification intensities was calculated, and the differences between the induced versus control condition are given. The Benjamini-Hochberg FDR method was used to compute multiple testing corrections for *P* values to determine whether the changes in protein abundance were significant. Download Table S5, XLSX file, 0.2 MB. Copyright © 2021 Aguilar Suárez et al. 2021 Aguilar Suárez et al. https://creativecommons.org/licenses/by/4.0/ This content is distributed under the terms of the Creative Commons Attribution 4.0 International license.

## DISCUSSION

### A protein synthesis boost in midi*Bacillus*.

The aim of our present study was to elucidate the main physiological adaptations in a genome-reduced midi*Bacillus* strain with enhanced capacity for the secretion of difficult-to-produce proteins. To this end, we analyzed the responses of both the parental strain 168 and the midi*Bacillus* strain upon induced production of the staphylococcal protein IsaA by label-free MS. This proteomics approach allowed the identification of ∼33% and ∼45% of the theoretical proteome of the parental and midi*Bacillus* strains, respectively. Importantly, even though the total numbers of quantified proteins were comparable for the two strains (see Table S2 in the supplemental material) and their core proteomes were similar (Fig. S1), substantial differences in the respective proteomic signatures were identified that revealed critical physiological alterations and a different adaptive behavior under conditions of protein secretion stress.

One of the most striking features of midi*Bacillus* concerned the high upregulation of proteins involved in protein synthesis and modification upon induced IsaA production (Fig. 4). This finding is particularly remarkable if one considers the fact that protein synthesis is one of the most “expensive” processes in the cell, consuming up to 40% of the cellular energy resources (40). In fact, proteins involved in translation and proteins that make up the ribosome do already represent the major bacterial proteome mass (4, 41). This upregulation of proteins with protein synthesis functions will, on the one hand, represent an energetic burden for midi*Bacillus*. On the other hand, it is presumably one of the main features of midi*Bacillus* that support its enhanced capacity for protein production. What exactly triggers the increased production of proteins involved in translation upon induced synthesis of IsaA is presently not entirely clear, but in any case, this means that the cells are limited neither in energy nor in amino acids. Probably, this response is not related to altered synthesis or degradation of the alarmone (p)ppGpp, since the levels of the GTP pyrophosphokinase RelA were not altered upon IsaA induction (42, 43). Importantly, the enhanced capacity for protein synthesis of midi*Bacillus* is reflected not only in its hardware for protein synthesis but also in the enhanced general translation rate as detected with our *bmrC*-GFP fusion module, which was not induced despite the presence of the inducer clindamycin. This implies that the translation rates remained high enough to preclude BmrB-induced antitermination of the *bmrC*-GFP expression. In contrast, a substantially higher induction of *bmrC*-GFP was detectable in the parental strain 168, but also in the intermediate strain IIG-Bs27-24. The latter implies that the improved translational capabilities of the midi*Bacillus* and mini*Bacillus* strains were acquired somewhere along the 43 consecutive genome reduction steps from strain IIG-Bs27-24 to the delivery of midi*Bacillus* (i.e., strain IIG-Bs27-47-24) (17, 19, 44). Notably, it is conceivable that the increased synthesis of proteins involved in translation upon induced IsaA synthesis by midi*Bacillus* could be compensated by the observed overall reduction in synthesis and abundance of proteins (Fig. 2) (45). This would imply a substantial rerouting of the cellular resources toward the translational machinery and IsaA production.

### A protein secretion “highway” in midi*Bacillus*.

Aside from an enhanced capacity for translation, we observed that midi*Bacillus* contains several Sec secretion machinery components at elevated levels compared to the 168 strain (Fig. 7). In particular, the enhanced level of the major signal peptidase SipS would be sufficient to explain the improved processing of the IsaA precursor in midi*Bacillus* (30, 46). However, the increased levels of other secretion machinery components, like Ffh, FtsY, SecA, and PrsA, could also contribute to the improved secretion of IsaA. Interestingly, several previous studies involving the overproduction of secretion machinery components in B. subtilis showed that overproduction of PrsA leads to enhanced posttranslocational folding of various heterologous secretory proteins (47). In contrast, improved protein secretion was not shown so far when the cellular levels of Ffh or FtsY were increased, suggesting that these proteins do not represent a limiting factor for protein secretion, at least in the 168 strain (15, 31).

The increased levels of different secretion machinery components compared to B. subtilis 168 seem to be a constitutive feature of midi*Bacillus*, because this increase was observed both upon IsaA induction and under the noninduced condition (Fig. 7). The only secretion machinery-related protein that was upregulated was Rnc (Table 1). Rnc is an RNase III, which functions in the processing and degradation of RNA molecules. Its homologue in S. aureus was shown to regulate the expression of extracellular proteins by affecting the level of RNAIII (48), but a similar function in secretion was thus far not reported in B. subtilis. Accordingly, the observed Rnc upregulation did not result in an increase in the number of proteins secreted by midi*Bacillus*. Thus, we do not know the reason why several secretion machinery components are present at elevated levels in midi*Bacillus*. One possibility is that this is partly related to the absence of the eight main extracellular proteases (20). While this could explain the increased levels of the extracytoplasmic proteins SipS and PrsA, which are subject to degradation by the wall protease A (WprA) (15, 49), it is a less likely explanation for the higher levels of the cytoplasmic Ffh, FtsY, and SecA proteins.

Lastly, while the Sec pathway of midi*Bacillus* seems to be enhanced, there are apparently fewer proteins competing for membrane passage via this route. Nevertheless, the native XynA protein was no longer detected upon IsaA induction, which suggests that there could be competition between XynA and the overproduced IsaA. If so, a further increase in the extracellular levels of heterologous proteins could perhaps be achieved by deletion of the *xynA* gene to minimize competition for the Sec pathway. Also, it is important to know that a previous study on the absolute quantification of membrane proteins in midi*Bacillus* revealed that substantial amounts of IsaA accumulate in the membrane of this strain (18). This suggests that, despite the elevated levels of Sec secretion machinery components in midi*Bacillus*, this strain’s Sec pathway is still not optimally equipped for protein translocation and/or precursor processing by signal peptidase (18, 50). This opens up the possibility to enhance the production of IsaA by increased expression of Sec components or signal peptidases (15, 30, 45).

### A redirected protein secretion stress response.

The canonical secretion stress response in B. subtilis 168 involves the CssRS-dependent induction of HtrA and HtrB, which is triggered by the accumulation of unfolded or poorly folded proteins in the cell envelope (33, 51). In addition, certain heterologous secretory proteins also trigger a LiaRS-dependent secretion stress response, probably due to membrane perturbations. Unexpectedly, HtrA and HtrB were detectable in midi*Bacillus* at elevated levels already under the noninduced condition, and the induction of IsaA did not lead to a further increase. Likewise, midi*Bacillus* contained elevated levels of the secretion stress sensor CssS. Unfortunately, the cognate response regulator CssR was not detected in the present MS analyses. However, since the *cssR* and *cssS* genes are located in an autoinducible operon, it is conceivable that the higher level of CssS is mirrored by a higher level of CssR, which could explain the elevated levels of HtrA and HtrB. In any case, the high-level expression of HtrA, HtrB, and CssS under the noninduced condition has not been observed before in B. subtilis, and it is thus one of the characteristics of midi*Bacillus*. This could, in fact, be another feature that aids in the efficient secretion of proteins by midi*Bacillus*, because HtrA and HtrB function in the removal or refolding of misfolded proteins that might interfere with integrity of the cell envelope. Yet, the LiaRS-dependently expressed LiaH protein was detectable as an ON protein upon IsaA induction, suggesting that the IsaA production does cause some membrane perturbation in midi*Bacillus* (19). In contrast, LiaH was not detected in the 168 strain upon IsaA induction. Together, these observations show that responses to secretion stress in midi*Bacillus* differ from the canonical secretion responses.

Interestingly, the present proteomics data suggest that induced IsaA expression has a relaxing rather than a stressful effect on midi*Bacillus*. Proteins negatively regulated by the stringent response were upregulated in midi*Bacillus* upon IsaA induction. Clearly, this response was not observed in the parental strain 168. Normally, the stringent response allows the cell to survive starvation or growth-limiting stresses, and it prevents the waste of cellular resources by decreasing cellular functions related with growth and reproduction (52). Thus, proteins belonging to the Pyr and PurR regulons, which are involved in pyrimidine and purine biosynthesis, respectively, are repressed by the stringent response (52). Nonetheless, the proteins belonging to both regulons were found to be upregulated upon IsaA induction in midi*Bacillus* (Fig. S2). Particularly, PurR, the negative regulator of the PurR regulon, was significantly upregulated in midi*Bacillus*, but this upregulation did not influence the elevated expression of proteins belonging to the PurR regulon. This implies that the increase in the level of PurR was not high enough to repress the PurR regulon. However, it could also mean that the cells used the produced nucleotides at a higher rate. In contrast, the observed upregulation of proteins belonging to the PyrR regulon cannot be related to changes in the PyrR regulator, suggesting that in this case the response is associated with an altered stringent response.

Another surprise was that midi*Bacillus* showed downregulation of the SigB, Spx, and PerR regulons upon IsaA induction, suggesting that the onset of IsaA production is even beneficial to the cells (Fig. 9 and Fig. S4). An opposite effect of IsaA induction was observed in the parental strain 168, where several SigB- and Spx-regulated proteins tended to be present at increased levels upon IsaA induction. In general, expression of the SigB regulon protects the cell against potentially lethal stresses, including but not limited to oxidative stress (53). Upregulation of the Spx regulon is associated with oxidative and cell envelope stresses (37), whereas the PerR regulon responds to peroxide stress. A previous study indicated upregulation of PerR-controlled genes in a Bacillus subtilis strain upon elevated levels of reactive oxygen species (54), which is the opposite of what we observed upon IsaA induction in midi*Bacillus*. Importantly, a connection between secretion stress and the Spx regulon was uncovered by Helmann and coworkers (55), who showed that the stabilization of Spx requires the YirB antiadaptor protein. Induction of YirB depends on the CssR response regulator in the induced, phosphorylated state. Accordingly, secretion stress in the 168 strain can lead to upregulation of Spx-controlled proteins, whereas we observed the opposite in midi*Bacillus*. Here, it should be mentioned that the *yirB* and *yuxN* genes involved in this pathway for Spx regulation are still present in midi*Bacillus* but that the respective proteins were detected in neither this strain nor the 168 strain.

Altogether, the present study shows how genome minimization has resulted in a rewired secretion stress response in midi*Bacillus*, a strain with an enhanced capacity for protein synthesis and secretion. In particular, the dissection of this strain’s proteome upon induced expression of the secretory IsaA protein, and the parallel analysis of the 168 strain under the same conditions, provides plausible explanations for the improved capacity for protein secretion of midi*Bacillus*. Clearly, the results also provide leads for further enhancement of midi*Bacillus*’s performance, including the provision of purines and pyrimidines that might become limiting, or the targeted modulation of the observed secretion stress responses in this organism. We are therefore confident that genome-engineered strains like midi*Bacillus* will become useful assets in the production of high-value proteins, be it for pharmaceutical or for biotechnological applications.

## MATERIALS AND METHODS

### Bacterial strains, plasmids, and culture conditions.

Bacterial strains and plasmids used in this study are listed in Table 1. All B. subtilis strains were grown in lysogeny broth (LB; Becton, Dickinson) at 37°C with continuous shaking at 250 rpm. Medium was supplemented with 2 μg ml^−1^ erythromycin and 20 μg ml^−1^ kanamycin when required. Both B. subtilis 168 and midi*Bacillus* carried the *spaRK* genes in the *amyE* locus, which was necessary for subtilin-induced expression of the staphylococcal protein IsaA from plasmid pRAG3::*isaA*, as previously described (18, 56). Of note, pRAG3::*isaA* encodes an IsaA protein with the signal peptide of the B. subtilis XynA protein instead of the native signal peptide of IsaA in order to direct efficient secretion via the B. subtilis Sec pathway (18).

For production of IsaA, the B. subtilis strains 168 and midi*Bacillus* carrying pRAG3::*isaA* were grown in LB medium supplemented with antibiotics for 18 h and, subsequently, diluted in 100 ml of fresh LB medium without antibiotics to an OD_600_ of 0.15. Culturing was continued until an OD_600_ of 0.9 was reached, which corresponded to the exponential growth phase. At this point, the expression of IsaA was induced by addition of 1% (vol/vol) subtilin-containing culture supernatant of B. subtilis ATCC 6633 (56). After continued culturing for 2 h, samples were collected for LDS-PAGE, immunoblotting, and proteomic analyses. Noninduced control cultures were treated in the same way. All induced and noninduced cultures were performed in triplicate.

### Preparation of extracellular and cellular proteome fractions.

For the proteomic analyses, the growth medium and cell fractions were separated by centrifugation at 8,500 × *g* for 20 min at 4°C. The supernatant was used for further enrichment of the extracellular proteins, and harvested cells were washed with 50 ml of TE buffer (20 mM Tris, 10 mM EDTA, pH 7.5). The washing step was repeated twice before resuspension in 1 ml of TE buffer. The cell pellets, containing the soluble and hydrophobic protein fractions, were stored at −80°C until further processing and enrichment of the membrane and cytosolic protein fractions.

### Preparation of extracellular protein fractions.

Proteins present in the growth medium fraction were enriched by primed affinity bead purification with StrataClean beads (Agilent) and subsequently eluted from the beads by LDS-PAGE as previously described in detail by Bonn and colleagues (57). Protein bands were excised from the gel, washed, and digested with trypsin solution (Promega). Subsequent peptide elution was carried out by ultrasonication. Peptides were quantified using the Pierce quantitative colorimetric peptide assay (Thermo Fisher Scientific) and desalted with ZipTip C_18_ tips (Merck).

### Preparation of membrane and cytoplasmatic protein fractions.

Cells were disrupted in a FastPrep24 instrument (MP Biomedicals) (3 times for 30 s each at 6.5 m s^−1^) with 5 min of cooling between each cycle. Cellular debris and beads were removed by centrifugation (20,000 × *g* for 5 min at 4°C). The resulting supernatant was designated the whole-cell extract, and its protein concentration was determined by the Bradford assay following the manufacturer’s protocol. An aliquot with a protein content of 3 mg was used as starting material for the preparation of the membrane fraction. Volumes were adjusted up to 1.5 ml of TE buffer prior to ultracentrifugation (170,000 × *g*, 1 h, 4°C). The resulting supernatant was stored at −20°C for further processing of the cytosolic protein fraction, and the corresponding pellet was used to enrich the hydrophobic fraction, as described previously (58). Briefly, the pellet was treated with 750 μl of high-salt buffer (10 mM EDTA, 1 M NaCl, 20 mM Tris-HCl) and incubated in an ultrasonic bath to detach it. The pellet was resuspended carefully with a pipette tip, and the tip was rinsed with an additional 750 μl of the same buffer. The resulting suspension was mixed at 8,000 × *g* for 30 min at 4°C in a rotator prior to ultracentrifugation (170,000 × *g*, 1 h, 4°C). The ultracentrifugation step was repeated, and two more ultracentrifugation steps were performed under the same conditions but with alkaline carbonate buffer pH 11 (10 mM EDTA, 100 mM Na_2_CO_3_, 100 mM NaCl) and tetraethylammonium bromide (TEAB; 50 mM).

The pellets containing the crude cell membrane extract were dried and resuspended in 25 μl of urea solution (6 M urea, 2 M thiourea). The pipette tip was rinsed with 25 μl urea solution, and samples were sonicated for 5 min before protein quantification by the Bradford assay. Twenty micrograms of crude membrane extract was used for protein digestion using S-trap columns (Protifi) according to the manufacturer’s instructions. Peptides were resuspended in 0.1% (vol/vol) acetic acid, quantified with the Pierce quantitative colorimetric peptide assay (Thermo Fisher Scientific), and desalted with C_18_ ZipTips (Merck).

The abovementioned supernatant containing the cytoplasmic protein fraction was thawed, and proteins were quantified by the Bradford assay. The same protocol as for the membrane fraction was followed for the cytoplasmic protein digestion.

### Liquid chromatography and mass spectrometric analysis.

The separation of peptides was carried out by liquid chromatography (LC) with an EASY-nLC II LC system (Thermo Fisher Scientific) and measured in an LTQ Orbitrap mass spectrometer (Thermo Fisher Scientific). Purified peptides (1 μg for the extracellular fraction and 5 μg for cytoplasmic and membrane fractions) were loaded onto in-house self-packed columns (inside diameter [i.d.], 100 μm; outside diameter [o.d.], 360 μm; length, 200 mm; packed with 3.0-μm Dr. Maisch Reprosil C_18_ reversed-phase material, ReproSil-Pur 120 C_18_-AQ) by the LC system with 10 μl of buffer A (0.1% [vol/vol] acetic acid) at a constant flow rate of 500 nl/min without trapping. The peptides were subsequently eluted using a nonlinear 180-min gradient from 1% to 99% buffer B (0.1% [vol/vol] acetic acid in acetonitrile) with a constant flow rate of 300 nl/min and injected online into the mass spectrometer. MS and tandem MS (MS/MS) data were acquired with an LTQ Orbitrap XL (Thermo Fisher Scientific). After a survey scan at a resolution of 30,000 in the Orbitrap using lockmass correction, the five most abundant precursor ions were selected for fragmentation. Singly charged ions, as well as ions without detected charge states, were not selected for MS/MS analysis. Collision-induced dissociation fragmentation was performed for 30 ms with a normalized collision energy of 35, and the fragment ions were recorded in the linear ion trap.

### Data processing.

Raw data were imported into MaxQuant (1.6.3.3) (59) incorporated with an Andromeda search engine. Database search was carried out against reversed B. subtilis 168 or IIG-Bs27-47-24 strain databases, with manually added IsaA, SpaR, and SpaK sequences, and with common contaminants added by MaxQuant (59). The parameters used for the database search were as follows: peptide tolerance, 4.5 ppm; minimum fragment ion matches per peptide, 1; match between runs was enabled with default settings; primary digest mode, trypsin; missed cleavages, 2; fixed modification, carbamidomethyl C (+57.0215); variable modifications, methionine oxidation (+15.9949), acetylation N, and K (+42.0106). Results were filtered for 1% false-discovery rate (FDR) on spectrum, peptide, and protein levels.

Data were processed with the Perseus software for further analysis (60). Proteins were filtered in each subproteome fraction according to the predictions by PSORTb v.3.0.2 and the UniProt database (61, 62). Proteins were considered for further analysis only if they had a minimum of two unique peptides per protein and if the proteins were quantified in at least two out of three biological replicates. Cell-wall-associated proteins were excluded from the analysis, because no specific enrichment of such proteins was performed. If quantified proteins were present in only one condition, they were added to the list of OFF and ON proteins. The relative quantification of both conditions was based on the label-free quantification (LFQ) intensities from MaxQuant (63). Mean values were calculated based on the biological replicates, and relative protein abundance was represented as log_2_ fold change. Quantified proteins that were significantly changing between conditions were determined with the Benjamini-Hochberg FDR method, which was used to compute multiple testing corrections for *P* values, with the following parameters: number of randomizations, 250; FDR, 0.05; and S0, 0.1. Principal-component analysis (PCA) was done with the web tool ClustVis (64) and was used to evaluate reproducibility based on experimental replicates using the normalized LFQ values. Venn diagrams were drawn with the InteractiVenn software tool (65). Box plot visualizations were created in Python 3.7.6 with the plotly package. Voronoi treemaps were built using the Paver software (Decodon GmbH) on the basis of the functional categories and regulon list of the SubtiWiki database (28). In order to compare the relative amounts of secreted extracellular proteins via the Sec pathway upon IsaA induction, we first used GP4 (Gram-Positive Protein Prediction Pipeline) (33) to predict the proteins that were secreted via this pathway. Then, the intensity of the quantified proteins secreted via the Sec pathway was summed and divided by the summed intensity of all quantified proteins in the extracellular fraction. This value was then determined for each strain upon IsaA induction.

### Determination of transcriptional activity.

The *bmrC*-GFP module used to assess the efficiency of translational activity in the midi*Bacillus* and IIG-Bs27-24 strains was introduced into these strains by transformation with the plasmid pRMC-5′bmrC-gfp. Since the genome-reduced strains carry the mannitol-inducible *comKS* cassette, transformation was performed by addition of 5% mannitol as described before (66). The PG10 strain carrying the *bmrC*-GFP module was constructed in the context of a previous study (18). Relative transcriptional activity was determined by live cell array analyses as described previously (29, 67). Briefly, overnight cultures were diluted 1:1,000 in 100 μl culture supplemented with 0.2 μg/ml clindamycin or H_2_O in 96-well flat-bottom microtiter plates (Greiner Bio-One). Subsequently, cultures were incubated at 37°C in a BioTek Synergy 2 plate reader, where readings of OD_600_ and GFP fluorescence (excitation, 485/20 nm; emission, 528/20 nm) were recorded every 10 min for 12 h. Background GFP fluorescence was determined from control strains not carrying the synthetic *bmrC*-GFP expression module and subtracted from the readings from the strains with the *bmrC*-GFP module. Finally, arbitrary transcriptional activity units (TAU) were calculated with the equation [GFP*^t^* – (GFP^*t* − 1^)]/OD_600_*^t^*, where *t* represents a specific time point and *t* − 1 represents the previous time point at which the measurements were recorded.

### Immunoblotting.

Protein samples were prepared as described previously and separated by LDS-PAGE (68). Before loading, samples were corrected to an OD_600_ of 2.0. Separated proteins were blotted onto a nitrocellulose membrane (Protran). Subsequent immunodetection of bound proteins was performed with polyclonal anti-SipS, anti-FtsY, anti-Ffh, anti-TrxA, anti-CssS, anti-SecA, anti-HtrB, anti-HtrA, and anti-PrsA antibodies raised in rabbits. Antibody binding was visualized using secondary antibodies labeled with IRDye800CW. The presence of IsaA was monitored with the human IsaA-specific antibody 1D9 that had been directly labeled with IRDye 800CW (25, 69). Fluorescence was recorded at 800 nm with an Odyssey infrared imaging system (LiCor Biosciences). Western blot images were analyzed with the ImageJ software to determine the relative abundance of the proteins in midi*Bacillus* compared with the parental strain 168 (70). Statistical analyses were performed with GraphPad Prism version 9. *P* values of <0.05 were considered to indicate statistical significance.

### Data availability.

All the mass spectrometry proteomics data have been deposited to the ProteomeXchange Consortium (http://proteomecentral.proteomexchange.org) via the PRIDE partner repository with the data set identifier PXD021841.
