# Supplementary material for: Redirected Stress Responses in a Genome-Minimized ‘midiBacillus’ Strain with Enhanced Capacity for Protein Secretion
Source: mSystems. 2021 Dec 14;6(6):e00655-21. doi: 10.1128/mSystems.00655-21 (PMC8670375; doi:10.1128/mSystems.00655-21)
Supplement: FIG S1 [file msystems.00655-21-sf001.pdf]

a

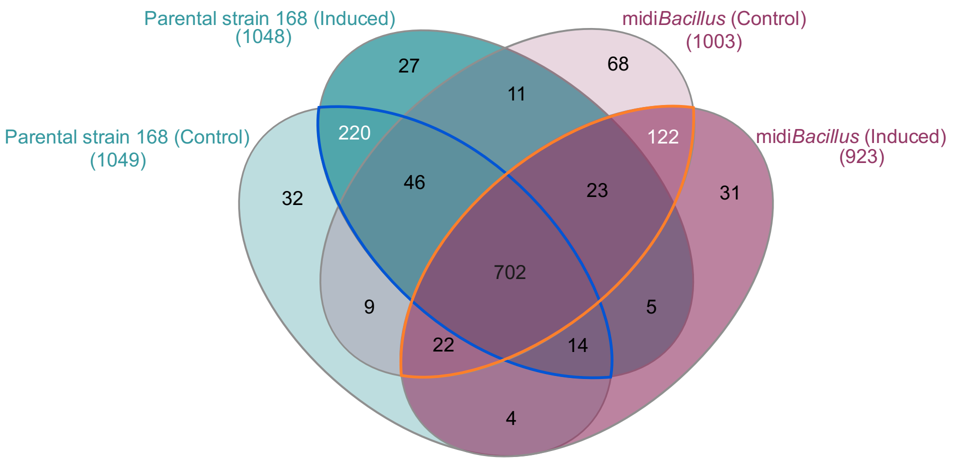

b

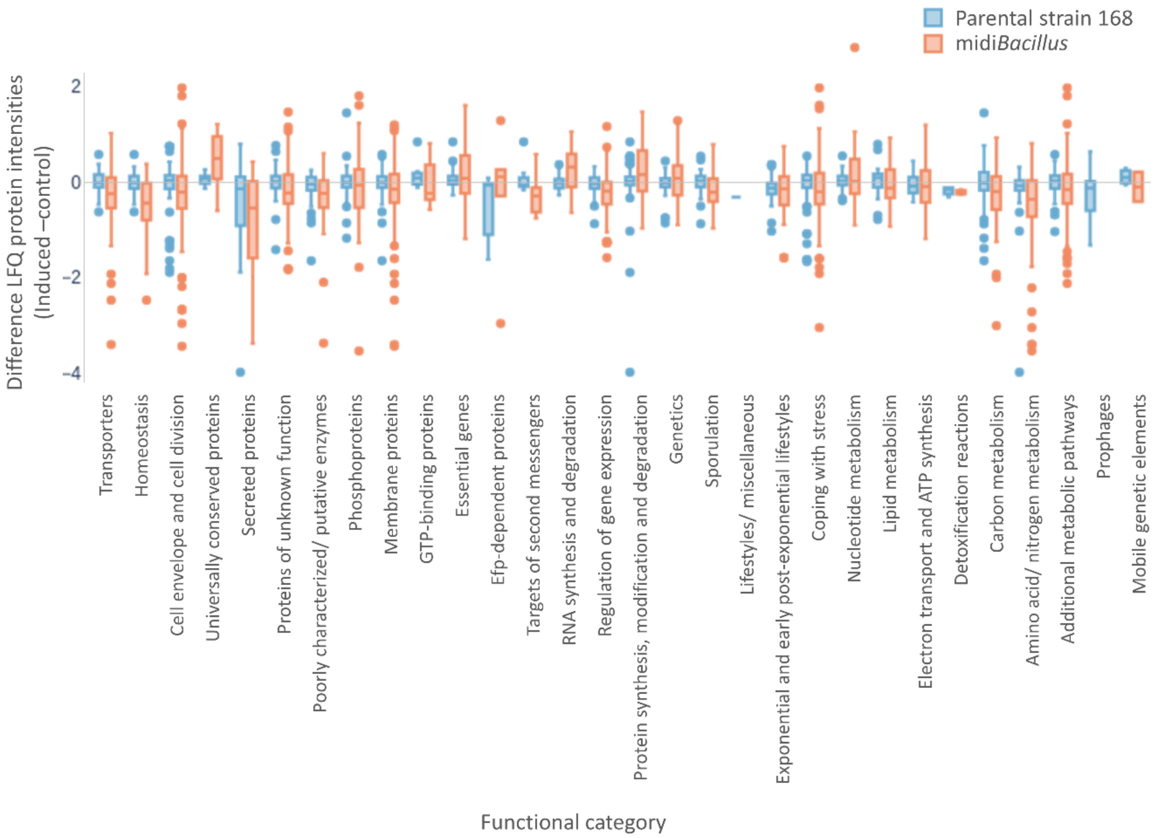

FIG S1 **Venn diagram of the quantified proteins in both strains and conditions and Box plot of shared proteins between conditions. (a)** Proteins were considered for quantification only if they were present in at least 2 of the 3 replicates and identified with a minimum of 2 unique peptides. White numbers indicate the quantified proteins that were detected in both conditions and exclusively in one strain. To note, both strains shared a core of 702 proteins that were expressed in both conditions. **(b)** Box plot of changes in the amounts of all quantified proteins present in both conditions per functional category. The changes correspond to the proteins in the overlapping region of the Venn diagram. For each protein, the difference was calculated as the amount based on LFQ values in the induced cells minus the values from the non-induced cells. Therefore, a positive value is the result of higher protein abundance in the induced condition than in the control. To note, some proteins belong to multiple functional categories. Outliers (values smaller or larger than the median ± 1.5 times the interquartile range) are plotted as points after the end of the whiskers, bars represent median values, the subdivision of boxes represents quartiles, and whiskers show the upper and lower quartiles. Elements depicted in blue correspond to the parental strain, while elements for midi*Bacillus* are depicted in orange.
