# Supplementary material for: Redirected Stress Responses in a Genome-Minimized ‘midiBacillus’ Strain with Enhanced Capacity for Protein Secretion
Source: mSystems. 2021 Dec 14;6(6):e00655-21. doi: 10.1128/mSystems.00655-21 (PMC8670375; doi:10.1128/mSystems.00655-21)
Supplement: FIG S2 [file msystems.00655-21-sf002.pdf]

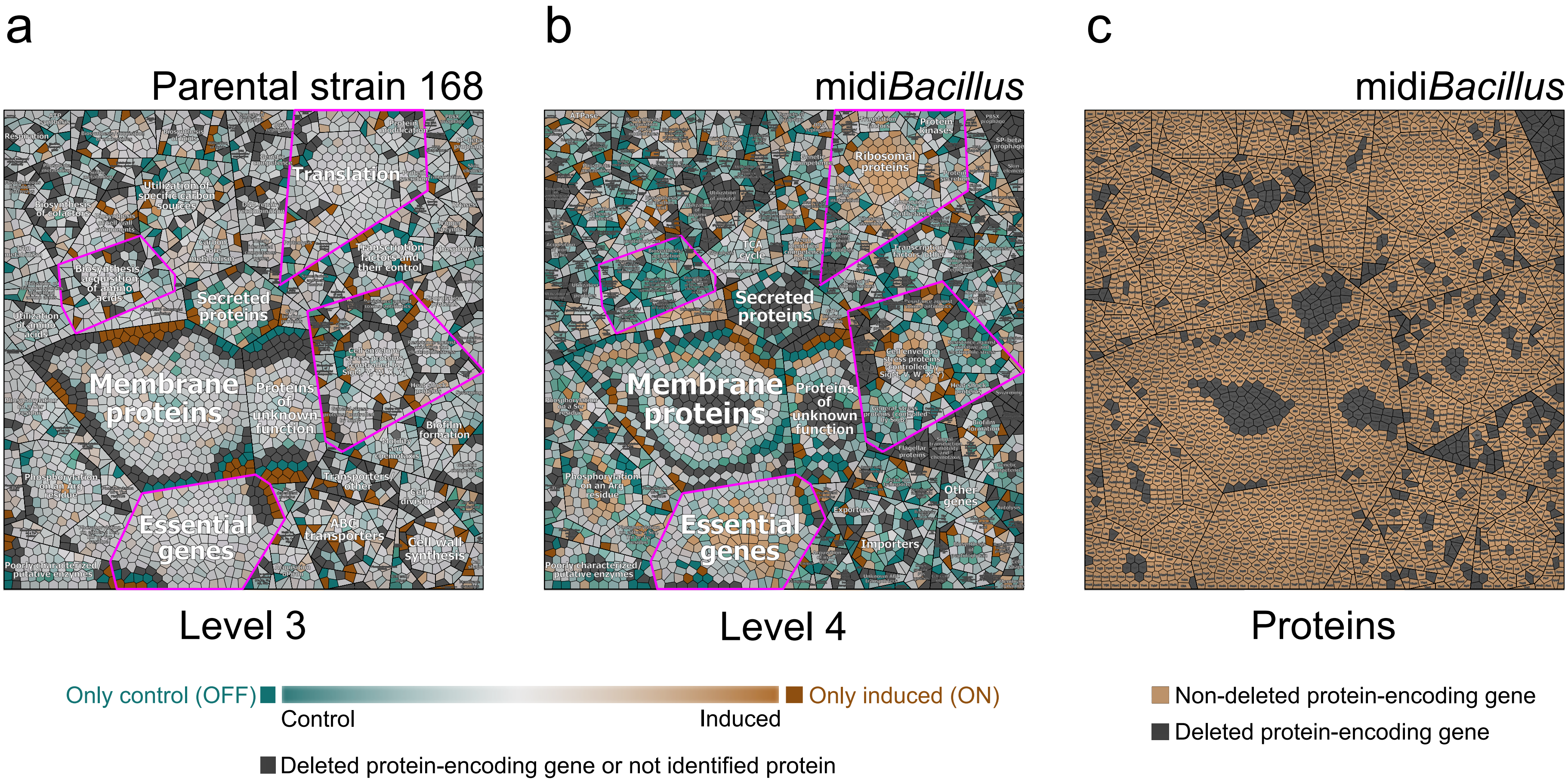

FIG S2 **Voronoi treemaps of the quantified proteins in the parental strain and midi*Bacillus* clustered per functional categories according to the SubtiWiki database.** The first panel shows the level three of the functional categories, the level four is indicated in the second panel, and protein names are presented in the last panel. Treemaps present the ratio of protein expression of the control versus the induced condition, for **(a)** the parental strain and **(b)** midi*Bacillus*, as colour code. Proteins coloured in shades of orange are more abundant in the induced condition; proteins coloured in shades of turquoise are more abundant in the control condition. Therefore, darker colours illustrate larger differences in protein expression. ON proteins (coloured in dark orange) are present only in the induced condition, while OFF proteins (coloured in dark turquoise) are present only in the control condition. In light grey colour are indicated the non-identified proteins in the panel a and the non-identified, or deleted protein-encoding genes in panel b. Relevant regulons are highlighted by fuchsia-coloured lining in panel a and b. **(c)** For all quantified proteins of the parental strain and the midi*Bacillus* strain, it is indicated whether the respective protein-encoding gene is deleted (in light grey colour) or present (light brown) from the midi*Bacillus* genome.
