# Supplementary material for: Redirected Stress Responses in a Genome-Minimized ‘midiBacillus’ Strain with Enhanced Capacity for Protein Secretion
Source: mSystems. 2021 Dec 14;6(6):e00655-21. doi: 10.1128/mSystems.00655-21 (PMC8670375; doi:10.1128/mSystems.00655-21)
Supplement: FIG S3 [file msystems.00655-21-sf003.pdf]

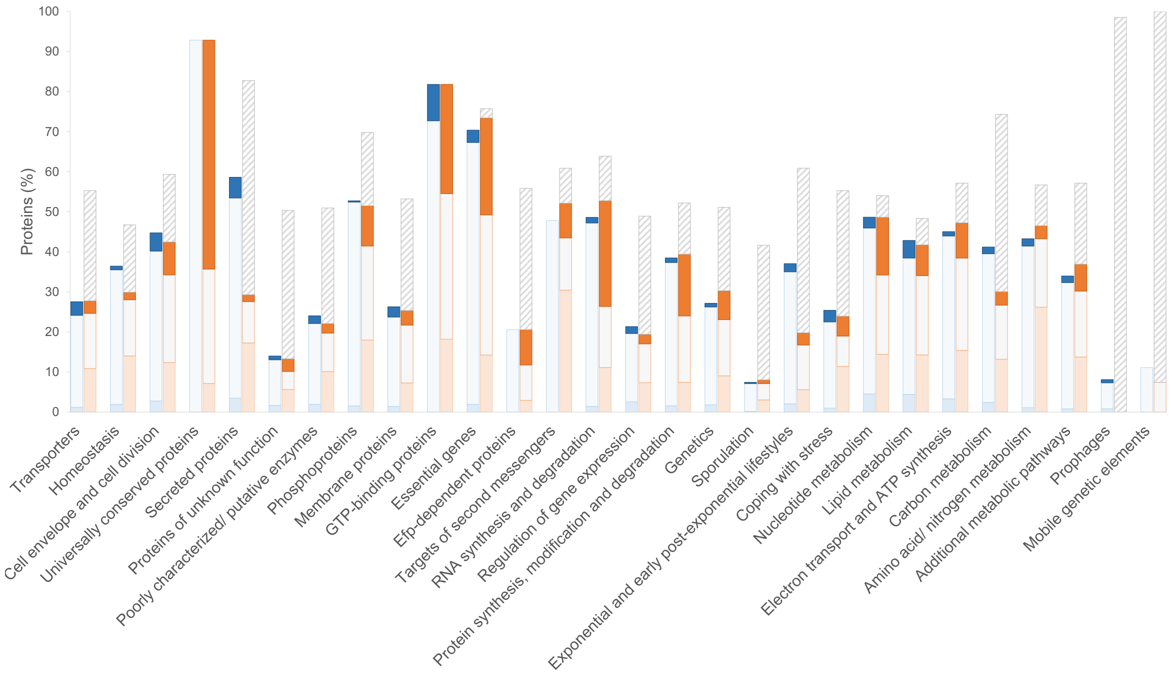

Deleted protein-encoding genes (midi*Bacillus*)

ON and significantly more abundant proteins (midi*Bacillus*)

Equally abundant proteins (midi*Bacillus*)

Significantly downregulated and OFF proteins (midi*Bacillus*)

Significantly upregulated and ON proteins (Parental strain 168)

Equally abundant proteins (Parental strain 168)

Significantly downregulated and OFF proteins (Parental strain 168)

FIG S3 Distribution of quantified proteins enriched per functional category. Percentages were calculated based on the total number of proteins in the parental strain per category according to the SubtiWiki database. Proteins in the non-induced control (OFF) and significantly downregulated proteins are represented in the lower part of the bar followed by the equally abundant proteins and, subsequently, the group of ON and significantly upregulated proteins per condition. Proteins whose genes were deleted in the midi*Bacillus* strain are indicated with light grey diagonal lines at the top of the bar. Non-identified proteins are not depicted for better visualization of the data, but their percentage can be inferred by subtraction of the depicted data from 100%. Of note, the higher total percentage of proteins identified for the midi*Bacillus* compared to 168 means that relatively more proteins of a particular category were identified for the midi*Bacillus*.
