# Supplementary material for: Redirected Stress Responses in a Genome-Minimized ‘midiBacillus’ Strain with Enhanced Capacity for Protein Secretion
Source: mSystems. 2021 Dec 14;6(6):e00655-21. doi: 10.1128/mSystems.00655-21 (PMC8670375; doi:10.1128/mSystems.00655-21)
Supplement: FIG S4 [file msystems.00655-21-sf004.pdf]

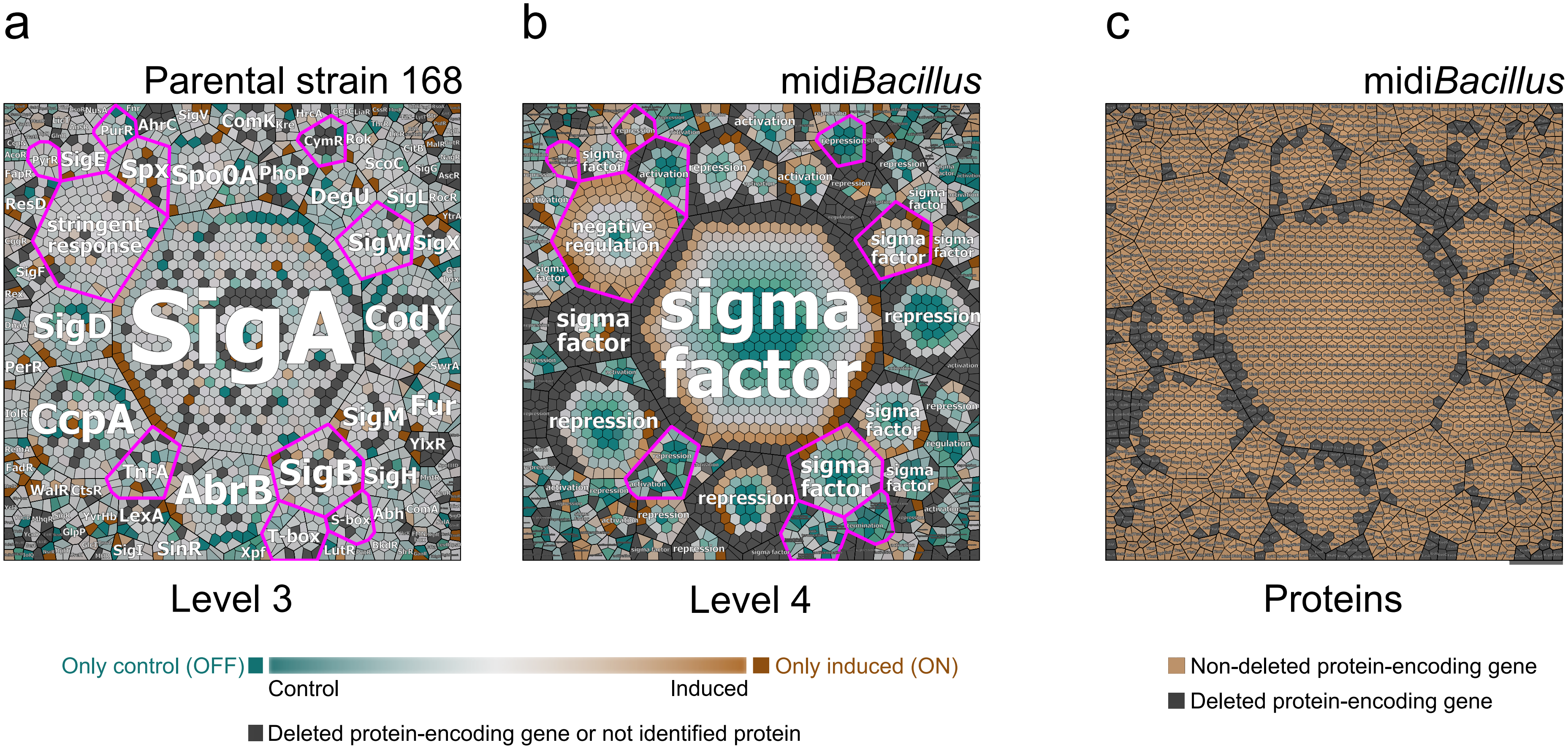

FIG S4 Voronoi treemaps of the quantified proteins in the parental strain and midi*Bacillus* clustered per regulons according to the SubtiWiki database. The first panel shows the regulator of each regulon, the mode of regulation is indicated in the second panel, and protein names are presented in the last panel. Treemaps present the ratio of protein expression of the control versus the induced condition, for **(a)** the parental strain and **(b)** midi*Bacillus*, as colour code. Proteins coloured in shades of orange are more abundant in the induced condition; proteins coloured in shades of turquoise are more abundant in the control condition. Therefore, darker colours illustrate larger differences in protein expression. ON proteins, which are present only in the induced condition, are coloured in dark orange; OFF proteins, which are present only in the control condition, are coloured in dark turquoise. In light grey colour are indicated the non-identified proteins in the panel a and the non-identified or deleted protein-encoding genes in panel b. Relevant regulons are highlighted by fuchsia-coloured lining in panels a and b. **(c)** For all quantified proteins in the parental strain and midi*Bacillus*, it is indicated if the respective protein-encoding gene is deleted (in light grey colour) or present (light brown) in the genome of midi*Bacillus*.
